# Pattern and risk factors of congenital anomalies in a pediatric university hospital, Alexandria, Egypt

**DOI:** 10.1186/s42506-018-0004-3

**Published:** 2019-01-09

**Authors:** Marwa Shawky Mohammed Abdou, Aida Ali Reda Sherif, Iman Mohamed Helmy Wahdan, Khaled Saad El din Ashour

**Affiliations:** 1 Department of Epidemiology, High Institute of Public Health, Alexandria University, Alexandria, Egypt; 2 General and Paediatric Surgery, Department of Pediatric Surgery, Faculty of Medicine, Alexandria University, Alexandria, Egypt

**Keywords:** Congenital anomalies, Risk factors, Prevalence, Children/pediatric, Egypt

## Abstract

**Background:**

Congenital anomalies (CAs) are structural, functional, or metabolic anomalies that originate during intrauterine life and can interfere with the body functions. In Egypt, the prevalence of CAs is increasing. The study aimed to estimate the frequency, describe the types, and identify the possible risk factors of CAs among infants attending the Pediatric University Hospital, Alexandria, Egypt.

**Methods:**

A retrospective case series and a case-control study were conducted. Patients’ records for the years 2010–2015 were reviewed, and a sample of 200 infants (100 cases and 100 controls) was taken from infants presented to Pediatrics, Pediatric Surgery, and Genetics Clinics of the hospital. Data were collected using a record review checklist and a predesigned interviewing questionnaire.

**Results:**

The study revealed that congenital anomalies of the digestive system (38.0%), musculoskeletal system (32.9%), and circulatory system (11.0%) were the most common types of CAs. Males were more affected with CAs than females (63% versus 37%). The major risk factors for CAs were old-aged parents, complications during pregnancy, unprescribed medications and excessive vitamin A intake during pregnancy, exposure to chemicals and pesticides during pregnancy, and living near mobile strengthening stations.

**Conclusion:**

Congenital malformations of the digestive, musculoskeletal, and circulatory systems were the most common types of CAs in the Pediatric Hospital. To prevent CAs, there is a need to restrict the prescription of medications that may have a teratogenic effect.

## Introduction

Congenital anomalies (CAs) are a worldwide problem. They are important causes of childhood deaths, chronic illness, and disability. The World Health Organization (WHO) estimated that annually, 303,000 newborns die within 4 weeks of birth worldwide due to CAs. The WHO defined CAs as structural, functional, or metabolic anomalies that originate during intrauterine life and can interfere with the body functions [1].They result from defective embryogenesis or intrinsic abnormalities in the development process [2].

Congenital anomalies are classified according to severity into major and minor anomalies [3]. They can also be classified into three groups of severity: minor, severe, and lethal anomalies. Severe and lethal anomalies together are considered as major anomalies [4].On the other hand, the international classification of diseases classified CAs according to the affected body system [5].

According to WHO (2015), about three million babies are born yearly with major CAs constituting about 3% of all newborns [1]. The global report of birth defects (2006) showed that the prevalence of CAs varied between high-, middle-, and low-income countries with 94% of all CAs occur in middle- and low-income countries. CAs were as high as 82/1000 live births in Sudan and as low as 39.7/1000 live births in France [6]. In 2006, the prevalence of CAs in the USA, UK, Germany, and Canada was between 45 and 50/1000 live births [6, 7]. In Africa and the Middle East, the reported prevalence was much lower. It ranged between 20 and 30/1000 live births in Kenya, Uganda, Nigeria, Saudi Arabia, and Pakistan [8–12]. In Egypt, the prevalence of CAs was 65.3/1000 live births in 2006 [6]. Within Egypt, the reported rates showed variations. They were the lowest in Assuit (20.6/1000) [13].

Congenital anomalies are a significant cause of infant morbidity and mortality. In 2006, worldwide, out of 2.68 million neonatal deaths, the WHO estimated that 11.3% of them died from CAs [2]. Approximately 95% of the children who died from CAs were from middle- and low-income countries [6]. In Egypt (2008), deaths from CAs constituted about 15% of all infant deaths [14]. Congenital anomalies can also result in long-term physical, mental, visual, and auditory disabilities if not managed appropriately and have significant negative impacts on individuals, families, health care system, and societies [1, 15].

The exact cause of CAs is unknown in about 40–60% of cases. Factors that may increase the risk of occurrence of CAs include genetic disorders, socioeconomic and demographic factors, nutritional factors including maternal obesity, infections during pregnancy, taking certain drugs, ionizing radiation, chemicals, and air pollution. Pregnancy-associated conditions such as insulin-dependent diabetes [2, 8], hypertension during pregnancy such as antepartum hemorrhage, twin pregnancy, oligohydramnios, and polyhydramnios were also found to be associated with more CAs [2, 13, 16, 17].

Some congenital anomalies can be prevented through removal of risk factors or reinforcement of protective factors. Important interventions include ensuring having healthy diet and maintain a healthy weight, ensuring adequate dietary intake of vitamins and minerals especially folic acid, avoiding harmful substances such as tobacco, avoiding infections known to be associated with CAs, and reducing environmental exposure to hazardous substances such as heavy metals and pesticides and exposure to certain medications and radiation [1].

The aim of this study was to estimate the frequency of CAs among infants presented to the Pediatric University Hospital in Alexandria, Egypt, in six consecutive years (2010–2015), to describe the nature of CAs among them, and to identify the possible risk factors of these anomalies.

## Methods

### Study setting

The study was conducted in the Pediatric University Hospital during the period November 2015–May 2016. This hospital is a tertiary care hospital drawing cases from Alexandria and nearby governorates.

### Study design

Two approaches: a retrospective case series and a case-control study were used.

### Sampling and data collection methods

All available inpatient and outpatient records of pediatrics, genetics, and pediatric surgery departments in the Pediatric University Hospital for the years 2010–2015 were reviewed. A record review checklist was used to collect information on sociodemographic data (age, sex, and residence), diagnosis, duration of stay in hospital, and condition at the time of discharge.

For the case-control study, the sample was calculated using Epi info7, 2015. Based on a power of 90%, a confidence interval 95%, a ratio of controls to cases of 1:1, a percent of consanguinity among controls of 35.3%, a percent of consanguinity among cases of 64%, and an odds ratio of 3.258 [13], the minimum required sample was 138 infants (69 cases and 69 controls). The sample was rounded to 200 infants (100 cases and 100 controls). The clinics were attended by the researchers 3 days a week for 6 months, and cases (infants with CAs) were consecutively recruited till reaching the required sample size. Controls (infants without CAs) matched for age were selected from the same departments till reaching the required sample size. A predesigned interviewing questionnaire was used to collect data from the mothers, and when not present, the following data were collected from the guardians of infants: sociodemographic data, maternal data (personal habits and obstetrics and gynecological history), family history, associated fetal factors (gestational age, mode of presentation, and birth weight), and diagnosis.

Identification of some risk factors such as environmental risk factors was dependent on reporting by the mother. Regarding excessive intake of vitamin A, it was defined as an intake of 10,000 IU of vitamin A or more assessed by the number of tablets taken [18]. The gestational age was divided into preterm (< 37 weeks), term (37–42 weeks), and post-term (> 42 weeks).

### Statistical analysis

The collected data were revised, coded, and analyzed using SPSS version 20 (IBM, USA). Abnormalities were classified based on the primary abnormality as defined under ICD 10. Descriptive statistics were calculated. Analytical statistics such as chi-square and odds ratio were done. Differences at *p* value < 0.05 were considered statistically significant. A stepwise logistic regression was carried out to explore the predictors of CAs. The variables entered in the model were the significant variables in the univariate analysis including sex, complications of pregnancy, medications during pregnancy, excessive vitamin A intake, exposure to pesticides, and living near strengthening mobile stations. Other variables that were thought to have a role in the occurrence of CAs such as consanguinity and family history of CAs were also entered in the model.

## Results

### Record review results

A total number of 5710 cases of CAs were recorded in El Shatby University Hospital in 6 years 2010–2015. Neonates (0–28 days) constituted 34% of the sample, and post-neonates (28 days to less than 1 year) constituted 66%. More than two thirds of cases (70.6%) were males, while females constituted only 29.4%. Concerning the residence of the infants having CAs, about half (48.2%) of the infants were from Alexandria while 51.8% were from other governorates (Table 1).

**Table 1:** Demographic characteristics of infants with CAs reported in the Pediatric University Hospital, Alexandria, Egypt, 2010–2015

Demographic characteristics	Frequency	Percent
Age		
Neonates	1935	34.0
Post-neonates	3775	66.0
Sex		
Male	4029	70.6
Female	1681	29.4
Residence		
Alexandria governorate	2755	48.2
Other governorates	2955	51.8
Total	5710	100.0

The duration of hospital stay ranged between 1 day and 66 days in the six studied years. The majority of infants (85%) stayed 1–10 days in the hospital, 9.9% of infants stayed 11–20 days, 3.4% of infants stayed 21–30 days, and 1.7% of infants stayed 30–66 days.

Based on data reported in the records, the majority of infants (82.9%) were well at the time of discharge, 14.6% of infants died, 1.9% of infants needed follow-up, and 0.6% of infants were ill. The number of infants discharged well increased throughout the studied years (from 73.3 to 88.8%). Those who died decreased from 19.7% in the year 2010 to 10.2% in the year 2015. Those who needed follow-up showed a more or less decreasing pattern.

Table 2 shows that among infants with CAs, 70.6% were males and 29.4% were females. As regards the different types of anomalies throughout the studied years, digestive system anomalies were the most frequent type of CAs constituting38.0%, followed by musculoskeletal anomalies (32.9%), circulatory system anomalies (11%), genetic disorders (8.4%), and genitourinary system anomalies (4.8%). Other CAs were less frequent, and multiple anomalies were rare (only 0.7%). Musculoskeletal and genitourinary system anomalies were more common in males while circulatory system anomalies were more common in females. There was a statistically significant difference between males and females regarding all types of congenital anomalies except genetic disorders.

**Table 2:** Frequency of different types of CAs by sex throughout the studied years in the Pediatric University Hospital, Alexandria, Egypt, 2010–2015

Type of CAs	Male		Female		Total		*Z* test	*p* value
	*N* = 4029 (70.6%)		*N* = 1681 (29.4%)		*N* = 5710 (100%)			
	Frequency	Percent	Frequency	Percent	Frequency	Percent		
Digestive system anomalies	1496	37.1	672	39.9	2168	38.0	2.01	0.043*
Musculoskeletal anomalies	1453	36.1	423	25.2	1876	32.9	7.99	0.000*
Circulatory system anomalies	364	9.0	265	15.7	629	11.0	7.40	0.000*
Genetic disorders	352	8.7	127	7.6	479	8.4	1.46	0.141
Genitourinary anomalies	229	5.7	48	2.9	277	4.8	4.53	0.000*
Other CAs	116	2.9	124	7.4	240	4.2	7.71	0.000*
Multiple anomalies	19	0.5	22	1.3	41	0.7	3.41	0.000*

*p < 0.05

**Fig. 1:**
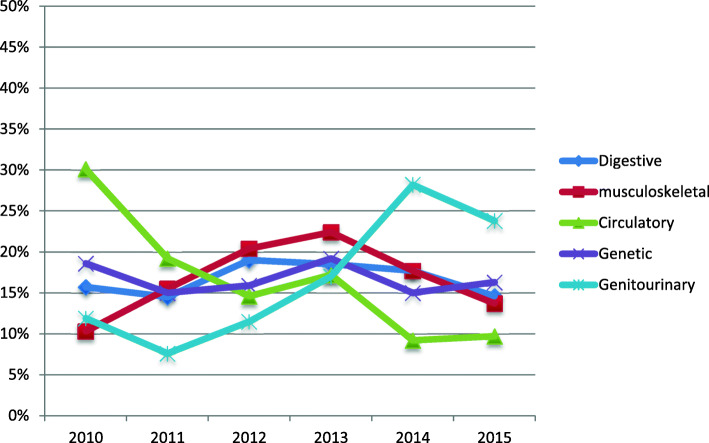
Pattern of the most common CAs in the Pediatric University Hospital, Alexandria, Egypt, 2010–2015

Regarding the pattern of the most common types of CAs in the different years, Fig. 1 shows that digestive system anomalies constituted around 15–19% of all types of anomalies with slight variations from 1 year to the other. Musculoskeletal anomalies constituted 10.3% of all types of anomalies in 2010. They increased in 2011, 2012, and 2013, then decreased in 2014 and 2015. Circulatory anomalies showed a decreasing pattern over the years. Genetic disorders constituted 18.6% of all types of CAs in 2010. They decreased in 2011 then increased in 2012 and 2013. They decreased again in 2014 and increased in 2015. Regarding genitourinary disorders, they constituted 11.9% of all types of CAs in 2010. They increased from 2011 to 2014 then decreased in 2015.

### Case-control study results

The characteristics of the studied infants are shown in Table 3. The percent of post-neonates was lower than neonates in the studied cases than in the controls (79% versus 91%); however, the mean age in days was nearly the same in the two groups. Males were more than females in cases (63% versus 37%), while females were slightly more than males in controls (53% versus 47%). There was a significant difference between males and females having CAs (*χ*^2^ = 5.172, *p* = 0.033). CAs were 1.92 times more likely to be among males than females (OR = 1.92). There were no much differences between the cases and controls with respect to the gestational age, mode of presentation, or delivery.

**Table 3:** Characteristics of the studied infants in the Pediatric University Hospital, Alexandria, Egypt, October 2015–May 2016

Characteristics of infants	Cases (*n* = 100)	Controls (*n* = 100)
	Percent	Percent
Age		
Neonatal	21	9
Post-neonatal	79	91
Mean age in days ± SD	127.47 ± 109.85	126.41 ± 91.18
Sex		
Male	63	47
Female	37	53
Gestational age		
Preterm (< 37 weeks)	10	10
Full-term (37–42 weeks)	86	83
Post-term (> 42 weeks)	4	7
Mode of presentation		
Cephalic presentation	74	67
Breech presentation	15	16
Complex presentation	2	7
Do not know	9	10
Mode of delivery		
Cesarean section	66	61
Normal delivery	33	39
Assisted delivery	1	0

Table 4 revealed that fathers of cases were slightly older than fathers of controls. The same was for the mothers, but the differences were not statistically significant. Fathers of cases had a higher percent of smokers, but the difference was not statistically significant. Regarding the consanguinity, 47% of cases and 38% of controls had positive consanguinity history between parents with no significant difference. Infants with CAs were 1.44 times more likely to have consanguinity history between parents compared to infants without CAs (OR = 1.44, CI = 0.824–2.541). Mothers of cases included more obese mothers than that of controls, more multipara, and multigravida, but the difference was not statistically significant. Oligohydramnios, hypertension, and preeclampsia were significantly more among mothers of cases of CAs. Taking unprescribed medications during pregnancy, excessive vitamin A intake, exposure to chemicals especially phenol and toluene, exposure to pesticides, and living near strengthening mobile stations were significantly higher among mothers of cases than controls (*p* < 0.05).

**Table 4:** Exposure to risks among the studied cases of CAs and their controls in the Pediatric University Hospital, Alexandria, Egypt, October 2015–May 2016

Risks	Percent in cases (*n* = 100)	Percent in controls (*n* = 100)	OR	95% CI (lower-upper)	Chi-square	*p* value
Paternal risks						
Age of father						
45 or more	12	4	3.27	(0.095–0.983)	4.348	0.065
Below 45	88	96				
Father’s cigarette smoking	58	48	1.50	(0.856–2.614)	2.007	0.202
Consanguinity						
Yes	47	38	1.44	(0.824–2.541)	1.657	0.252
No	53	62				
Maternal risks						
Age of mother						
35 or more	18	9	2.22	(0.192–1.058)	3.468	0.097
Below 35	82	91				
Obesity (BMI ≥ 30)	35.3	23.9	1.74	(0.821–3.681)	2.108	0.187
Number of deliveries						
More than one	82	71	1.86	(0.954–3.631)	3.365	0.095
One	18	29				
Number of pregnancies						
More than one	87	77	2.00	(0.948–4.215)	3.388	0.097
One	13	23				
Ovulation support	12	9	1.38	(0.554–3.434)	0.479	0.645
Twin pregnancy	7	3	2.43	(0.611–9.694)	1.684	0.331
Oligohydramnios	15	3	5.71	(1.597–20.386)	8.791	0.005**
Hypertension	26	9	3.55	(1.568–8.048)	10.009	0.003**
Preeclampsia	7	0	2.08	(1.793–2.402)	7.254	0.01**
Polyhydramnios	9	4	2.37	(0.706–7.977)	2.057	0.251
Anemia	46	40	1.28	(0.729–2.239)	0.734	0.475
Unprescribed medications	36	17	2.75	(1.416–5.327)	9.267	0.004**
Excessive vitamin A intake	46	12	6.25	(3.041–12.834)	28.072	0.000**
Family history of CAs	39	26	1.82	(0.998–3.319)	3.852	0.07
Environmental risks						
Exposure to radiation	6	4	1.53	(0.419–5.603)	0.421	0.748
Exposure to chemicals	28	10	3.50	(1.595–7.679)	10.526	0.002**
Exposure to pesticides	53	33	2.29	(1.291–4.059)	8.160	0.006**
Living near strengthening mobile stations	10	2	5.44	(1.161–25.521)	5.674	0.033*
Living near high electricity transmission towers	9	4	2.37	(0.706–7.977)	2.057	0.251

*p < 0.05; **p < 0.01

**Fig. 2:**
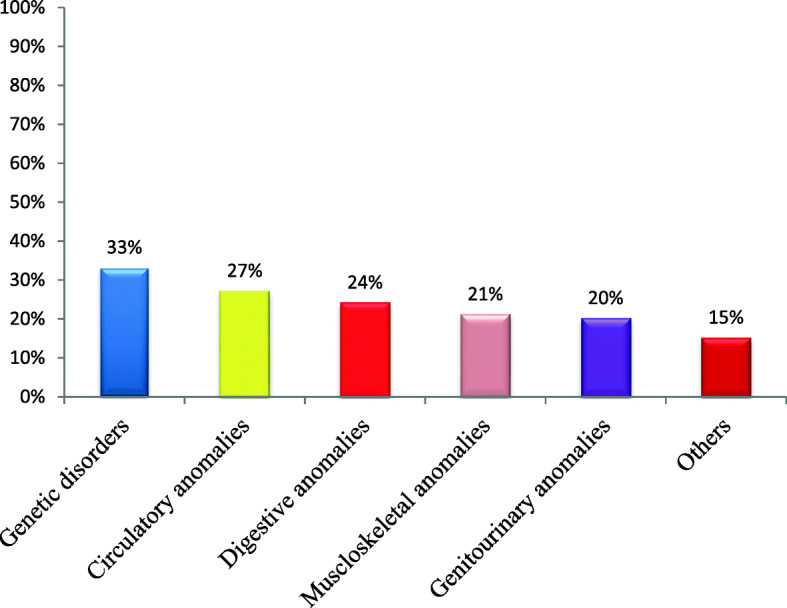
Types of CAs of the studied infants in El Shatby University Hospital, Alexandria (October 2015–May 2016)

The most common types of CAs among the studied cases were genetic disorders (33%), cardiovascular anomalies (27%), digestive system anomalies (24%), musculoskeletal anomalies (21%), genitourinary anomalies (20%), and other anomalies (15%) (Fig. 2).

A stepwise logistic regression was done for the determinants of CAs. Eight independent variables were used to build the stepwise logistic regression model, namely sex, consanguinity, complications of pregnancy, medications during pregnancy, excessive vitamin A intake, exposure to pesticides, living near strengthening mobile stations, and family history of CAs. Five variables were significantly affecting the occurrence of CAs: complications of pregnancy (OR = 7.559), medications during pregnancy (OR = 6.722), excessive vitamin A intake during pregnancy (OR = 23.068), living near mobile strengthening stations (OR = 4.154), and exposure to pesticides during pregnancy (OR = 5.435) (Table 5).

**Table 5:** Stepwise logistic regression analysis results for the determinants affecting the presence of CAs among infants in the Pediatric University Hospital, Alexandria, Egypt, October 2015–May 2016

Independent variables	Coefficient *B*	*p* value	Odds ratio	95% confidence interval
Complications of pregnancy	− 0.988	0.006	7.559	0.184–0.753
Medications during pregnancy	− .0999	0.010	6.722	0.173–0.784
Excessive vitamin A intake	− 1.910	0.000	23.068	0.068–0.323
Strengthening mobile stations	− 1.699	0.042	4.154	0.036–0.937
Exposure to pesticides	− 0.775	0.020	5.435	0.240–0.884
Constant	1.848	0.000		

Overall percentage of the model was 72.5%

## Discussion

The present study showed that among infants with CAs, 70.6% were males and 29.4% in females. These results were in agreement with a retrospective study conducted in El Shatby University Hospital [19] which found that males admitted to the surgery unit of the hospital during the years 2009–2011 were more than females.

In the current study, digestive system anomalies and musculoskeletal anomalies constituted 38.0% and 32.9% of the total CAs respectively. They were more than those recorded in a surveillance study conducted in Glasgow and Clyde in 2015–2016 where GIT anomalies constituted only 9% while musculoskeletal anomalies constituted 25% [20]. Different rates might be attributed to the fact that the present study was a hospital-based case series while the other study was conducted using a population-based surveillance program.

Circulatory system anomalies constituted 11% of the total CAs in the current study. The surveillance study conducted in Glasgow and Clyde found that the prevalence of circulatory system anomalies was 13.9% [20], and the EUROCAT records showed even higher figures for circulatory system anomalies (35%) [21]. Genetic disorders and genitourinary anomalies constituted 8.4% and 4.9% of total CAs, respectively, in the studied years. The percent of genitourinary anomalies was much lower than that of the EUROCAT records (24.2% of total CAs) [21]. The difference between the rates might be due to the difference in the methodology used and in exposure to various risk factors.

The case-control study revealed that males were 1.92 times at higher risk of having CAs than females. This result was in agreement with Tennant et al., who found a 15% increased risk of CAs in male infants than female infants in North England (1985–2003) [22].

The current study showed that children with CA were more among mothers aged above 35 years than mothers aged less than 35 years, but this was not statistically significant. A study done in Tanzania in 2013 [23] and a study done in Ain Shams University, Cairo, in 1995–2009 [14] showed a significant association between mothers aged above 35 years and CAs.

The present study showed that infants born to fathers aged 45 years or more were more than those having fathers aged below 45 years. In Knudsen, Danish in 1980–1996, there was no significant association between paternal age and the overall prevalence of CAs but there was a significant association between paternal age and limbs’ anomalies, multisystem syndromes, and Down syndrome [24]. Another study done in California, 1989–2002, showed that there was a significant association between paternal age and some selected CAs [25].

Obese females were at a higher risk of having infants with CAs. Mills et al., in New York 1993–2003 [26], found a strong association between obesity and CHDs. A systematic review concluded a contribution of obesity to certain types of CAs such as NTDs, CHDs, and orofacial defects [27].

The results of the present study agree with other studies that found a high frequency of CAs in infants born to females with a history of oligohydramnios. Oligohydramnios interferes with fetal movement resulting in a cascade of developmental events leading to fetal anomalies [28]. The same observation was seen in the case of polyhydramnios. Some studies indicated that CAs may be a potential cause for polyhydramnios but not the reverse. Some anomalies like oesophageal atresia, duodenal atresia, and some neuromuscular disorders impair the swallowing reflex and increase urine and amniotic fluid production resulting in polyhydramnios [29].

Mothers of children with CAs that gave history of hypertension during pregnancy were significantly more than mothers of the control group. A systematic review by Ramakrishnan et al. (2015), found an association between maternal hypertension and CVS anomalies. The association between treated hypertension and CVS anomalies was stronger than that for untreated hypertension which might suggest that antihypertensive drugs lead to an additional increase in the risk of CAs [30]. There was also a twofold increased history of preeclampsia among mothers of cases in the present study. Nelson et al., in Texas (2012), showed an increased rate of CAs in preeclampsia and suggested that fetal anomalies related to preeclampsia are limited to isolated microcephaly and hypospadias, and both microcephaly and hypospadias are associated with impaired fetal growth in females with preeclampsia [31]. There was also a one and half fold increased risk of CAs in infants born to females who experienced antepartum hemorrhage in the current study. Shawky et al. revealed an association between early antepartum hemorrhage in pregnancy and CAs [14].

Mothers of cases that gave history of taking different types of unprescribed medications were more than mothers of controls. Different studies showed an association between different types of drugs and CAs such as Uziel and Rozental [32] who showed that antiepileptic drug (AED) administration resulted in CNS anomalies and Etemad et al. [33] who found a high frequency of major anomalies, heart defects, and hypoplasia of the midface and fingers, known as anticonvulsant embryopathy, in infants exposed to anticonvulsant drugs in utero mainly AEDs. Yakoob et al., meta-analysis, suggested an increased risk of CVS anomalies, orofacial clefts, and NTDs following intake of oral beta-blockers in the first trimester of pregnancy [34].

History of exposure to chemicals during pregnancy was three and a half folds more among mothers of cases than mothers of controls. Desrosiers et al. [35], in the USA, observed an increased prevalence of NTDs among infants born to women exposed to chlorinated solvents during the peri-conceptional period, but they did not find an association between orofacial defects and exposure to chlorinated solvents. Hjortebjerg et al., in Denmark in the period 2001–2003, observed a positive association between exposure to paint fumes in the first trimester of pregnancy and the risk of CNS and ear, face and neck, and renal system anomalies [36].

Mothers of children with CAs indicated exposure to pesticides during pregnancy much more than mothers of controls. This result agrees with Kielb et al. [37], who found an elevated risk of having an infant with isolated gastroschisis among mothers aged 20 years old or more occupationally exposed to pesticides and with Liete et al. [38] who also found an association between exposure to pesticides and CAs in newborns. On the other hand, Clementi et al. [39] reported that there is no evidence that exposure of mothers to pesticides during pregnancy has any effect on the prevalence of CAs.

The present study showed that there was a significant association between living near strengthening mobile stations and the presence of CAs. Most of the human studies focused on the effects of mobile radiations and mobile base stations on the overall health of human beings. Very limited studies focused on the effect of mobile phones and mobile strengthening stations on the embryo development and CAs. The non-thermal interaction may lead to an increase in the production of reactive oxygen species leading to DNA damage in the sperms [40].

### Limitations of the study

Mothers of children with CAs, in their efforts to detect the reasons of the CAs, may be more prone to remember exposure to risk factors than mothers of normal children. In addition, some of the severe CAs may be missed as the newborn may die soon after birth, and some of the mild CAs may not be detected early after birth such as deafness and so the pattern of the type of CAs may not reflect the full picture.

Although it would have been better to calculate the prevalence of CAs, yet it was difficult to obtain the data of the denominator that is why the researchers calculated the frequency of CAs.

Diagnosis of risk factors was reporter dependent, and there was also a lack of standardized measurement of some factors such as oligohydramnios.

## Conclusions and recommendations

The current study showed that digestive system anomalies, musculoskeletal anomalies, circulatory system anomalies, and genetic disorders were the most common types of CAs in the Pediatric University Hospital. The major risk factors for CAs were old-aged parents, occurrence of complications during pregnancy, unprescribed medications and excessive vitamin A intake during pregnancy, exposure to chemicals and pesticides during pregnancy, and living near mobile strengthening stations.

There is a need for screening of females in preconception and peri-conception periods as well as for neonatal screening to early detect and treat CAs. Obstetricians should restrict the prescription of medications that may have a teratogenic effect. They should regularly assess pregnant females by ultrasound for early detection of CAs. Public awareness should be raised about the importance of folic acid in preventing CAs and the dangers of exposure of pregnant females to environmental hazards.
